# Preparation and characterization of ZnO/graphene/graphene oxide/multi-walled carbon nanotube composite aerogels

**DOI:** 10.3389/fchem.2022.992482

**Published:** 2022-08-15

**Authors:** Yang Shen, Zhihao Yuan, Fengjiao Cheng, Zhen Cui, Deming Ma, Yueyue Bai, Shuqing Zhao, Jieyao Deng, Enling Li

**Affiliations:** ^1^ School of Science, Xi’an University of Technology, Xi’an, China; ^2^ School of Electrical Engineering, Xi’an University of Technology, Xi’an, China; ^3^ School of Automation and Information Engineering, Xi’an University of Technology, Xi’an, China

**Keywords:** ZnO, graphene, multi-walled carbon nanotube, aerogels, photocatalytic

## Abstract

ZnO/Graphene (G)/Graphene Oxide (GO)/Multi-walled Carbon Nanotube (MCNT) composite aerogels with a three-dimensional porous structure were prepared by the sol-gel method under average temperature and alkaline conditions, combined with freeze-drying process and heat treatment process. The photocatalytic degradation of Rhodamine B (RhB) was mainly studied. The scanning electron microscope (SEM) test results showed that the morphology uniformity of the ZnO/G/GO/MCNT composite aerogel was significantly enhanced, which effectively solving the agglomeration problem of MCNT and ZnO. The photocatalytic degradation test results of RhB show that due to the synergistic effect of physical adsorption and photocatalytic degradation, the total degradation efficiency of RhB by ZnO/G/GO/MCNT could reach 86.8%, which is 3.3 times higher than that of ZnO. In addition, the synergistic effect of ZnO and G effectively hinders the recombination of photo-generated electron-hole pairs and enhances photocatalytic activity. The ZnO/G/GO/MCNT composite aerogel can be applied in the visible light catalytic degradation of water pollution.

## Introduction

In recent years, with the rapid economic development and the increasing environmental pollution (Cui et al., 2020), the management of organic pollutants in water bodies has attracted great concern (Tang et al., 2022). Traditional methods of treating organic contaminants in wastewater include physical adsorption (Cui et al., 2022a; Das et al., 2022; Wang et al., 2022), chemical oxidation (Li et al., 2021; Yan et al., 2021; Zhang et al., 2021), and biological degradation (Sun K et al., 2021; Marciano et al., 2021; Zou et al., 2021). Compared with traditional methods, photocatalytic technology, as a new “green sustainable technology,” features the advantages of environmental protection, high efficiency, low energy consumption (Shen et al., 2016), no secondary pollution, and a wide range of applications, etc. It has rapidly emerged as a research hotspot for academics and industry in various countries. Meanwhile, many methods of photocatalyst surface modification modification such as adsorption, doping, and compounding have also emerged (Cui et al., 2019; Zhang and Cui, 2022).

The photocatalytic oxidation reaction uses semiconductors, such as titanium oxide (TiO_2_) (Gopinath et al., 2020; Li et al., 2020), zinc oxide (ZnO) (Kegel et al., 2018), vanadium oxide (VO_2_) (Zhu et al., 2018), molybdenum disulphide (MoS_2_) (Yin et al., 2018; Sun and Schwingenschlögl, 2021a), etc., as catalysts (Sun and Schwingenschlögl, 2020). Under light irradiation, the electrons in the valence band (VB) of the semiconductor are excited and shifted to the conduction band (CB). The holes in the VB capture electrons from the hydroxyl groups in the surrounding environment, generating free radicals with strong oxidizing properties (Sun and Schwingenschlögl, 2021b; Shen et al., 2022a). The free radicals degrade the organic matter adhering to the surface of the semiconductor into carbon dioxide and water, thus achieving efficient purification of organic pollutants (Sun M.et al., 2021).

ZnO is the II-VI direct bandgap novel inorganic semiconductor material (Shen et al., 2022a). It has high photocatalytic activity (Yang et al., 2004; Sun C et al. 2018), cheap availability, and abundant reserves. So its application (Wang et al., 2018) for efficient photocatalysis (Yan et al., 2017) is one of the hottest research topics at present. However, due to the small size effect, ZnO can achieve high photocatalytic efficiency only at the nanoscale size. For nanoscale ZnO, it has a large specific surface area and high specific surface energy, and it is easy to agglomerate by itself. It has a strong surface polarity, which makes it difficult to disperse uniformly in the medium, resulting in few active sites for the photocatalytic oxidation of ZnO. In addition, ZnO exhibits the characteristics of a wide bandgap, whose bandgap is about 3.37 eV at room temperature (Shen et al., 2022b). As a result, it has poor electrical conductivity and a tendency to compound photo-generated electron-hole pairs, resulting in low quantum efficiency and a narrow response range to the solar spectrum. Moreover, although using metals or organic materials as loading materials for ZnO may compensate for these deficiencies, photo corrosion effects cause the loading materials to decompose and the shedding of catalytic particles, which may result in secondary pollution to the environment. These factors significantly limit the photocatalytic performance of ZnO. Therefore, solving the problems of complex dispersion, poor electrical conductivity, low solar light utilization, and severe photo corrosion of nanoscale ZnO is the challenge to achieve a significant increase in ZnO photocatalytic efficiency and improved cycling stability.

Graphene oxide (GO) has a high specific area. Its surface is rich in hydrophilic functional groups, and GO has a good affinity for dye molecules. So it is easy to achieve uniform dispersion of GO and adsorption of organic dye molecules dissolved in water. Removal of hydrophilic functional groups from the surface of GO, thereby reducing GO to graphene (G), can improve the electrical conductivity of the material.

It is reported that using G as a carrier for ZnO can improve its photocatalytic properties. Due to the good electrical conductivity of G, it creates a shallow potential Schottky barrier at the contact interface between ZnO and G. This reduces the compounding of photo-generated carriers in ZnO, thus improving the photocatalytic performance of ZnO. Sun et al. (2019) compounded ZnO with G. They found that this compound enabled photo-generated charge carrier transfer and effectively hindered electron-hole pair recombination, degrading 95.9% of methylene blue dye under UV irradiation conditions for 60 min. Pant et al. (2013) prepared Ag/ZnO/Reduced Graphene Oxide (RGO) composites, they found that it has suitable photocatalytic and antibacterial activities. The efficiency of perfect recovery and recycling of the catalyst after the reaction remains unchanged. In addition, Ahmad et al. (2018) combined nickel oxide and RGO to enhance the photocatalytic activity of the metal oxides. The formation of P-N heterojunctions and the strong interaction between nickel oxide and RGO dictated a high separation efficiency of photo-generated electrons and holes, enhancing the photodegradation activity of methylene blue dyes. However, the methods reported in these studies require UV light to achieve the photocatalytic reaction, indicating the need for severe conditions of use. Efficient photocatalytic reactions under visible light require more in-depth research.

Aerogels are nanomaterials with micro, mesoporous and microporous multilevel fractal network structures (Shen et al., 2019), which also can provide a good support skeleton for semiconductors. Besides, it can provide a special contact interface for photocatalytic reactions and accelerate the diffusion of photoelectrons in water (Li and Zhang, 2022).

Here, we have developed a method for assembling ZnO, G, GO, and Multi-Walled Carbon Nanotube (MCNT) into composite aerogels with a three-part porous structure. The composite material allows the nano-ZnO to be uniformly dispersed in water, reducing the compounding of photo-generated carriers in the ZnO, enhancing the electrical conductivity, and enabling efficient photocatalytic reactions to be developed under visible light conditions.

## Experimental

### Materials

The reagents used in this paper include Zinc acetate dihydrate, sodium hydroxide (NaOH), and Rhodamine B (RhB), which are all analytically pure and purchased from Sinopharm Chemical Reagent Co. Ltd., MCNT and GO are purchased from Suzhou Tanfeng Graphene Technology Co. Ltd., as shown in Table 1.

**TABLE 1:** The chemical reagent for preparing the sample.

Reagent name	Chemical formula	Specification
Zinc acetate dihydrate	C_4_H_6_O_4_Zn·2H_2_O	Analysis pure
Sodium hydroxide	NaOH	Analysis pure
Multi-walled Carbon Nanotubes (MCNT)	C	>95wt%
Graphene Oxide (GO)	—	>98wt%
Rhodamine B (RhB)	C_28_H_31_ClN_2_O_4_	Analysis pure

### Preparation of composite aerogels

**FIGURE 1:**
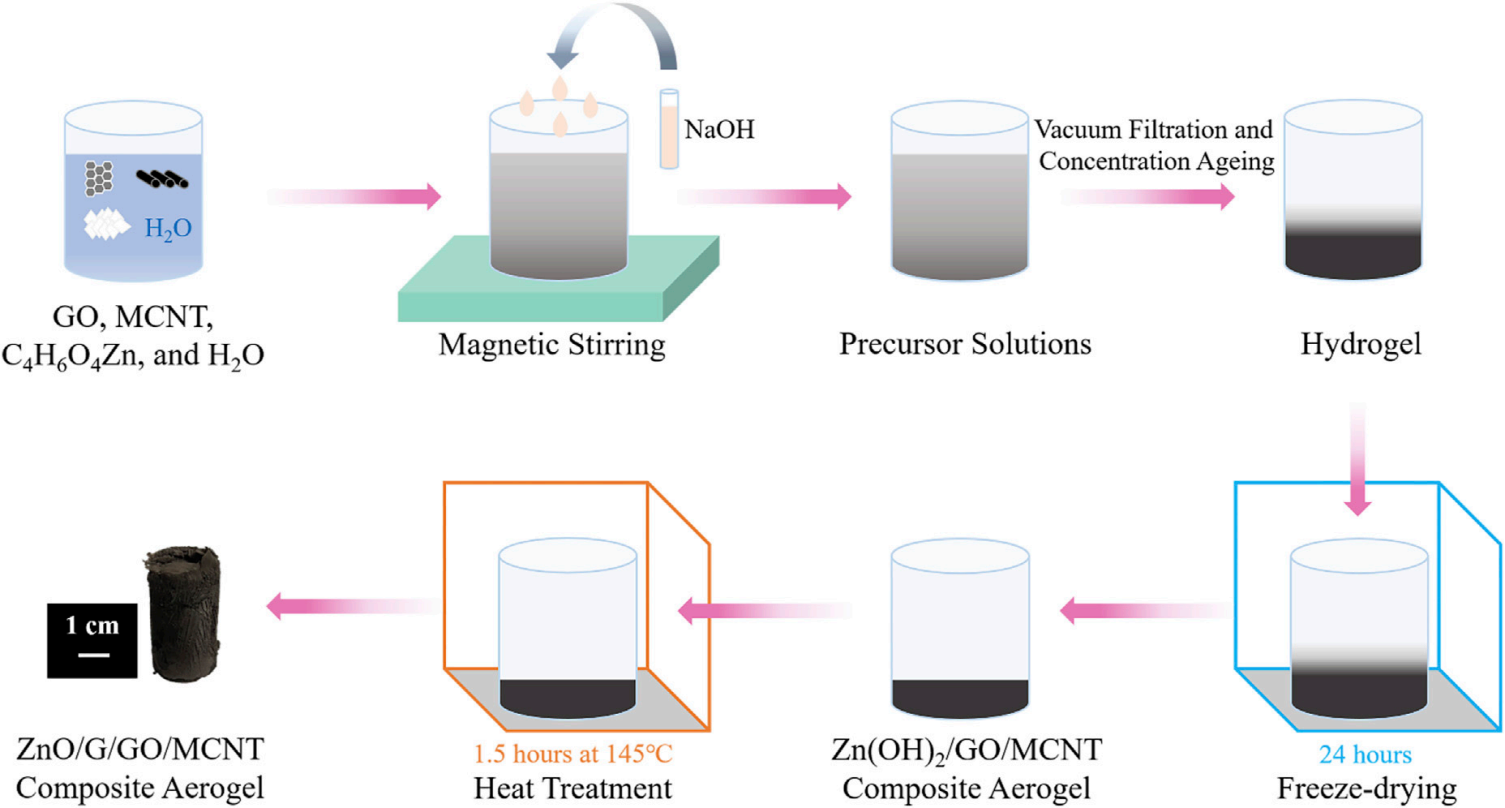
The preparation process of ZnO/G/GO/MCNT composite aerogel.

The preparation process of ZnO/G/GO/MCNT composite aerogel is shown in Figure 1. GO aqueous solution, MCNT aqueous solution, zinc acetate dihydrate solution, and distilled water mixed well at room temperature and pressure. Then, add NaOH solution in 4 equal parts, once at an interval of 1 min, 5–10 ml each time, with a drop acceleration rate of 5–10 drops per second, stirring continuously at room temperature to make the alkali fully react with the mixed solution. And precursor sols were obtained. The precursor sols were vacuum filtered to achieve hydrogels. Next, the hydrogel is placed in a freeze dryer for 1–2 h to ice entirely, then vacuum freeze-dried for 24 h to produce a Zn(OH)_2_/GO/MCNT composite aerogel. Finally, the Zn(OH)_2_/GO/MCNT composite aerogel is heat-treated in a thermostat or tube furnace for 1.5 h at 145°C. The Zn(OH)_2_ decomposes to ZnO, while GO is partially restored to G, obtaining ZnO/G/GO/MCNT composite aerogel.

To accurately describe the properties of ZnO/G/GO/MCNT composite aerogels, Different sample ratios are shown in Table 2. ZnO (sample #1), ZnO/MCNT composite aerogel (sample #2), and ZnO/G/GO/MCNT composite aerogel (sample #3).

**TABLE 2:** The raw material ratio of aerogel.

Sample #	6 mg/ml MCNT (ml)	6 mg/ml GO (ml)	1 mol/L C_4_H_6_O_4_Zn (ml)	1 mol/L NaOH (ml)
1	0	0	2.5	4 × 0.625
2	40	0	2.5	4 × 0.625
3	20	40	2.5	4 × 0.625

### Characterization

The micrograph of samples was characterized by a scanning electron microscope (SEM, Japan Electronics Corporation, JSM-6700F). X-ray diffractometer (XRD, Shimadzu Japan, XRD-7000) was used to describe the internal atomic or molecular structure of samples. TG-DTA curve of the material using a comprehensive thermal analyzer (Beijing Hengjiu, HCT-3). UV-Visible spectrophotometer (Unico, United States, UV-2355) is used to measure the absorbance of samples at wavelengths between 200 and 800 nm.

The wavelength-absorbance curve is plotted, and the absorption edge is estimated by the tangent method. Using monochromatic light of 200–800 nm to irradiate the composite material, obtaining the light absorption of composite material at different wavelengths. The bandgap (*E*_g_) is calculated as follows (Sauer et al., 2002):

$$E_{g}=\frac{hc}{\lambda_{m}}$$ (1)

where *h*, *c*, and *λ*_m_ denote Planck’s constant, speed of light in a vacuum, and maximum wavelength of absorbed light, respectively. *λ*_m_ can be estimated from the UV-Vis absorption spectrum. The absorption efficiency of composite aerogels for sunlight can be obtained by calculating the proportion of wavelengths less than *λ*_m_ in the solar range.

The photocatalytic activity of the resulting composite aerogel was investigated by the rapid degradation of RhB under visible light radiation. Firstly, 20 mg of the samples were added into a 100 ml range quartz beaker, and add 50 ml of RhB staining solution at a concentration of 10 mg/L. Secondly, by the water bath method, maintain a speed of 1,000 r/min at 30°C with a heated magnetic mixer to mix well. At the same time, the dark reaction under shade for 30 min to reach adsorption equilibrium. After that, 5 ml of solution from the dark reaction system was taken with a pipette for testing. Then, it was switched on a 350 W xenon cold light source for light reaction, and the light source 13.5 cm from the bottom of the beaker. 5 ml of solution is removed from the photoreaction system every 5 min with a pipette for testing. After six extractions, the test samples were centrifuged, and the absorbance of the supernatant was measured.

According to the Beer-Lambert law, the absorbance (*A*) of a dye solution is proportional to its concentration. In addition, the dye removal rates (*D*) are computed as follows (Cui et al., 2022b):

$$D=\left(1-\frac{C}{C_{0}}\right)\times 100\%=\left(1-\frac{A_{t}}{A_{0}}\right)\times 100\%$$ (2)

where *C*_0_, *C*, *A*_0_, and *A*_t_ represent the initial concentration of the fuel solution, the concentration of the dye solution after *t* minutes, the initial absorbance of the dye solution, and the absorbance of the dye solution after *t* minutes, respectively. The value of *A*_t_/*A*_0_ can be calculated from the relative absorption intensity of the UV-Vis absorption spectrum.

The photocatalytic degradation process of dyes mainly consists of mass transfer and photocatalytic reactions. The kinetic constants of photocatalytic reaction degradation are described by the Langmuir–Hinshelwood model as follows (Sauer et al., 2002):

$$R=-\frac{dC}{dt}=\frac{\kappa KC}{1+KC}$$ (3)

where *R*, *κ*, *K*, and *C* represent the total reaction rate of the dye at time *t*, the Langmuir rate constant, the equilibrium constant for the adsorption and desorption of the paint on the catalyst, and the concentration of the dye at time *t*, respectively. The Eq 3 can be simplified to a primary reaction kinetic model when the concentration of the paint is relatively low. The calculation is as follows (Cui et al., 2022b):

$$\ln\frac{C_{\text{0}}}{C}=\kappa Kt=k_{\mathrm{app}}t$$ (4)

where *k*_app_ indicates the primary reaction rate constant. Linear fit of ln (C_0_/C) and *t*, and the slope is *k*_app_, which can be used to characterize the photocatalytic reaction efficiency quantitatively.

## Results and discussion

### Morphology and compositional characterization

**FIGURE 2:**
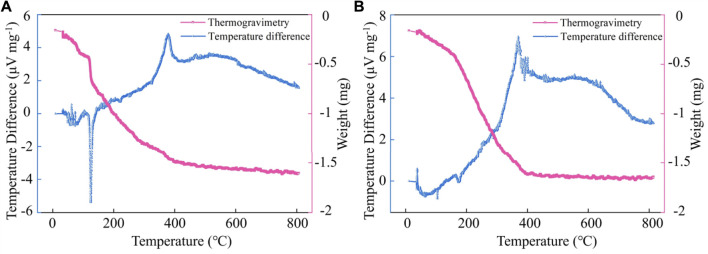
The TG and DTA curves of ZnO (sample #1) before **(A)** and after **(B)** 1.5 h heat treatment.

To accurately describe the thermal decomposition of Zn(OH)_2_, the Thermogravimetry (TG) and Temperature difference (DTA) curves of sample #1 before and after heat treatment are shown in Figures 2A,B, respectively. From Figure 2A, It can be observed that an endothermic peak appears at 124°C, which is mainly due to the absorption of heat by the thermal decomposition of Zn(OH)_2_, and the overall weight of the sample is decreasing. From Figure 2B, No vigorous endothermic or exothermic peaks were observed in the range of 0–150°C. Altuntasoglu et al. (2010) performed TG-DTA analysis on Zn(OH)_2_. They found an endothermic peak appeared at 134°C, and the final decomposition product was ZnO. Related reports are consistent with our results. Therefore, it proved that Zn(OH)_2_ in the Zn(OH)_2_/GO/MCNT composite aerogel could be converted entirely into ZnO after heat treatment at 145°C.

**FIGURE 3:**
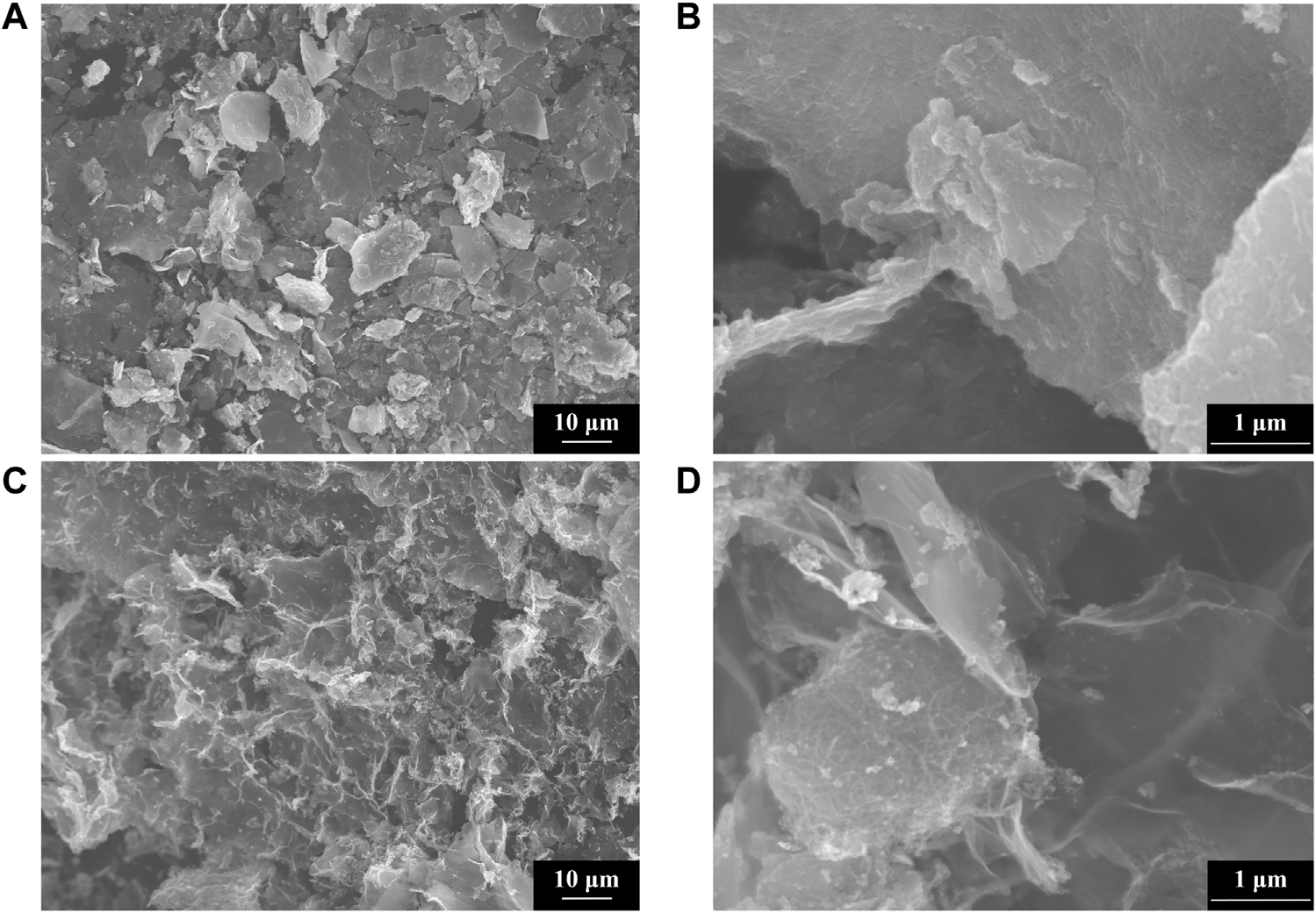
SEM of ZnO (sample A) at magnifications of **(A)** 1,000 and **(B)** 20,000 times. SEM of ZnO/G/GO/MCNT composite aerogel (sample C) at a multiplicity of **(C)** 1,000 and **(D)** 20,000 times.

The Scanning Electron Microscope (SEM) of sample #1 is shown in Figures 3A,B, respectively. Figure 3C,D exhibit the SEM of sample #3, respectively. By Figures 3A,C, it can be observed that the morphology uniformity of sample #3 was enhanced after adding graphene and Multi-walled carbon nanotubes. The dense adhesion of Multi-walled carbon nanotubes and ZnO particles to graphene sheets was observed under the SEM graph of Figures 3B,C, and ZnO/G/GO/MCNT composite aerogel (sample #3) effectively solved the clustering problem of ZnO.

**FIGURE 4:**
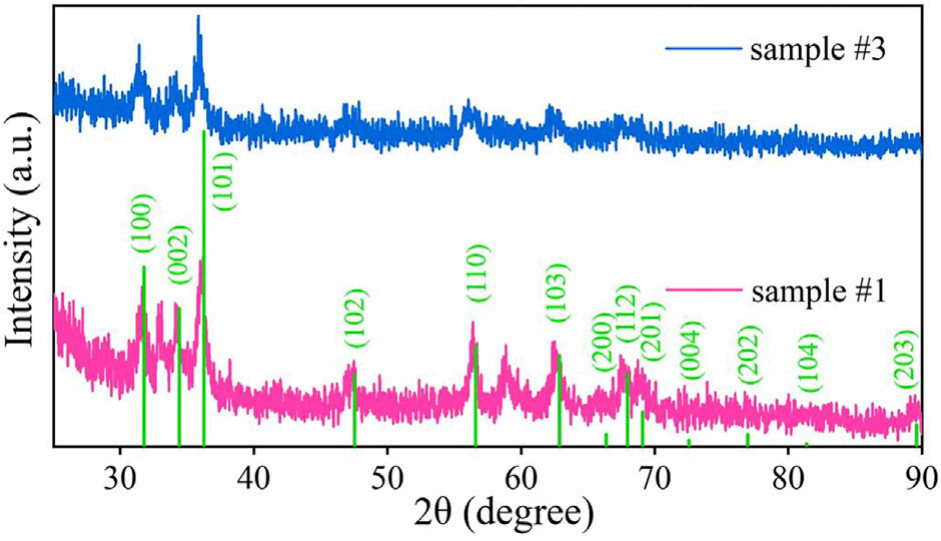
XRD images of ZnO (sample #1) and ZnO/G/GO/MCNT composite aerogel (sample #3). The green vertical line represents the peak position corresponding to the ZnO standard XRD standard card.

The XRD patterns of samples #1 and #3 are shown in Figure 4. The test range is 2θ = 25–90°. It can be observed that sample #3 has prominent narrow characteristic peaks at 31.42°, 34.2°, and 36.04°. Compared with the ZnO standard card, it is found that the corresponding crystal planes are (100), (002), and (101). In addition, broad characteristic peaks appear at 47.62°, 55.72°, 62.72°, and 67.58°, corresponding to the (102), (110), (103), and 112) crystal planes, respectively. It is verified that Z (OH)_2_, in sample #3, has been completely converted into ZnO. It is consistent with the results of the TG-DTA analysis.

### Optical absorption and photocatalytic degradation of RhB

**FIGURE 5:**
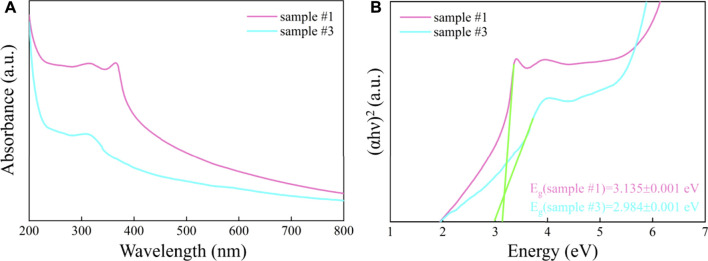
**(A)** The UV-Vis spectrum and **(B)** the relation between Kubelka-Munk function and photon energy of ZnO (sample #1) and ZnO/G/GO/MCNT composite aerogel (sample #3).

To demonstrate the change in the response range of the ZnO/G/GO/MCNT composite aerogel to the solar spectrum, we further studied the UV-Vis absorption spectra of sample #1 and sample #3, which are shown in Figure 5A. The relation between the Kubelka-Munk function and photon energy is shown in Figure 5B. For sample #1, the tangent (extrapolation method) calculates that the absorption edge is around 396 nm. For sample #3, its absorption edge is at 416 nm. Meanwhile, the band gap of samples #1 and #3 are calculated to be 3.135 and 2.984 eV, respectively. The reduction of the band gap may be due to the adsorption between different materials (Sun M et al., 2018; Cui et al., 2021a; Cui et al., 2021b). This will improve the photocatalytic degradation of pollutants by the composite material under visible light conditions.

**FIGURE 6:**
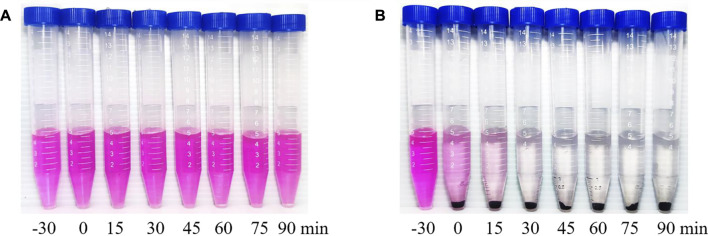
Experimental results of photocatalytic degradation of RhB by **(A)** ZnO (sample #1) and by **(B)** ZnO/G/GO/MCNT composite aerogel (sample #3).

The photocatalytic efficiency is the focus of our inquiry (Cui et al., 2022b). Sample #3 was subjected to photocatalytic experiments, and its photocatalytic activity was evaluated by comparing its photocatalytic degradation rate to RhB. The experimental results are shown in Figure 6B. −30–0 min is the dark reaction stage, and the light reaction starts from 0 min. To reach the experimental results, the same photocatalytic efficiency experiment was applied to sample #1 (Figure 6A). For sample #3, photocatalytic experiments proved that RhB achieved efficient degradation after 30 min of visible light irradiation.

**FIGURE 7:**
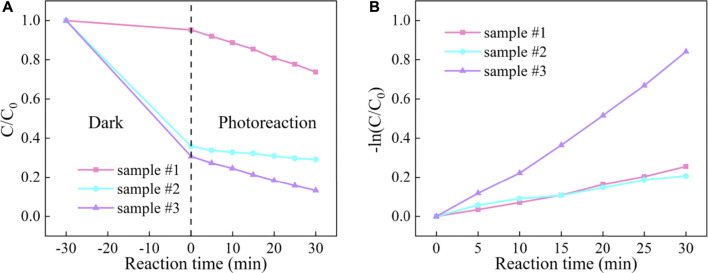
**(A)** RhB degradation curve and **(B)** reaction kinetic curve under visible light for ZnO (sample #1), ZnO/MCNT composite aerogel (sample #2), and ZnO/G/GO/MCNT composite aerogel (sample #3).

**FIGURE 8:**
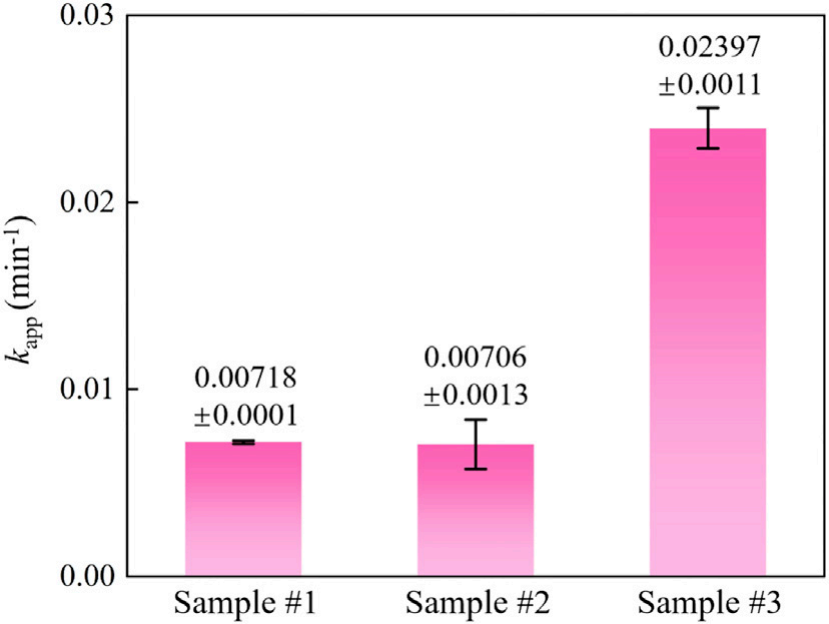
Reaction kinetic constant histogram for ZnO (sample #1), ZnO/MCNT composite aerogel (sample #2), and ZnO/G/GO/MCNT composite aerogel (sample #3).

The reaction curves and photocatalytic kinetics curves of different samples are depicted in Figures 7A,B, respectively. The results indicated that RhB adhered to the sample surface by adsorption within 30 min of the dark reaction phase until the adsorption and desorption reached equilibrium. At this stage, it mainly relies on the van der Waals forces between molecules to reach equilibrium quickly, and the adsorption or desorption process is physical adsorption. Besides, compared with sample #1, the physical adsorption is enhanced due to the introduction of MCNT, G, and GO. In the photoreaction stage, after 30 min, the degradation efficiency of ZnO (sample #1) was only 26.3%, and the degradation effect was the worst. The degradation efficiency of ZnO/MCNT composite aerogel (sample #2) was 70.8%, and the degradation effect was good. The degradation efficiency of ZnO/G/GO/MCNT composite aerogel (sample #3) is as high as 86.8%, and the degradation effect is the best. Cui et al. (2022b) reported a degradation rate of 99.4% at 90 min for g-C_3_N_4_/MoS_2_ composites. Similar results can be obtained based on the trend of our results. In addition, it can be observed from Figure 7B that the degradation rate of sample #3 accelerates from the photoreaction for 10 min, and it is also the fastest overall among all samples. Figure 8 more intuitively compares the kinetic reaction constants of each sample. It can be observed that the reaction kinetic constant of ZnO/G/GO/MCNT (sample #3) is 0.02497, which is larger than that of ZnO (0.00718, sample #1) and ZnO/MCNT composite aerogel (0.00706, sample #2). In conclusion, compared with single ZnO, the physical adsorption effect, photocatalytic degradation efficiency, and photocatalytic degradation rate of the ZnO/G/GO/MCNT composite aerogels were significantly improved. And it have higher application value.

## Conclusion

ZnO/G/GO/MCNT composite aerogels with a three-dimensional porous structure were prepared by the sol-gel method under average temperature and alkaline conditions, combined with a freeze-drying and heat treatment process. The photocatalytic degradation of RhB was mainly investigated. The SEM test results show that the morphology uniformity of the ZnO/G/GO/MCNT composite aerogel is significantly enhanced, effectively solving the agglomeration problem of MCNT and ZnO. TG-DTA analysis and XRD pattern showed that Zn(OH)_2_ was wholly decomposed into ZnO after heat treatment. The physical adsorption capacity of ZnO/G/GO/MCNT for RhB is significantly improved. And its total degradation efficiency can reach 86.8%, which is 3.3 times higher than that of ZnO (26.3%), indicating that the ZnO/G/GO/MCNT composite aerogels have high photocatalytic activity. In addition, the synergistic effect of ZnO and G can also enable photogenerated carrier transfer to hinder electron-hole pair recombination, enhancing the photocatalytic activity effectively. Therefore, the ZnO/G/GO/MCNT composite aerogel can be applied in the visible light catalytic degradation of water pollution.

## Data Availability

The original contributions presented in the study are included in the article/supplementary material, further inquiries can be directed to the corresponding authors.
